# Supplementary material for: Effectiveness of Booster Dose of Anti SARS-CoV-2 BNT162b2 in Cirrhosis: Longitudinal Evaluation of Humoral and Cellular Response
Source: Vaccines (Basel). 2022 Aug 8;10(8):1281. doi: 10.3390/vaccines10081281 (PMC9415026; doi:10.3390/vaccines10081281)
Supplement: Supplementary file 1 [file vaccines-10-01281-s001.zip › vaccines-1792956-supplementary.pdf]

Supplementary Method

Flow Cytometry analysis

Specifically, 2uL of PepTivator stock solution were used for antigen stimulation, 2 uL of CytoStim as positive control and 2 uL of sterile water/10% DMSO solution as negative control. After 2 hours of stimulation, Brefaldin A was added to each well for inhibiting the transport of proteins to the cellular membrane. Afterwards, the cells were incubated at 37°C in 5% CO2 for additional 4 hours, harvested and stained with fuorochrome-conjugated monoclonal antibodies as reported in **Supplementary Table S1** for the detection of the expression of surface activation induced markers in CD4 and CD8 T cells subset. The Viobility 405/452 Fixable Dye master mix (cat. 130-109-812, Mitenyi Biotec.) was also included for identifying the fraction of total living cells. SARS-CoV-2–reactive T cells were analysed after gate exclusion of doublets, debris, and dead cells as well as negative fraction of CD14+ and CD20+ cell subsets. After pre-gating on CD3+, activated CD4+ T cells were defined as activation of CD40 ligand (CD154+) and TNFα+, while activated CD8+ T cells were reported as TNF-α + and IFN-γ + as reported in **Supplementary Figure S1**. Following background subtraction of unstimulated cultures, negative values were set to zero. The threshold for positivity was set by calculating the 75th percentile minus the median of the values obtained. The flow cytometry analyses were performed on MoFlo Astrios cell sorter (Beckman Coulter) and FACS Canto2 (Becton Dickinson). The FlowJo (Becton Dickinson) and GraphPad-Prism 8.4.3 software were employed for the visualisation and statistical analyses of data.

SARS-CoV-2–binding B cells and plasma cells were identified as CD3-CD19+GFP+ and CD3-CD19+CD38highGFP+ respectively after gate exclusion of doublets, debris, and dead cells and following background subtraction of B cells interacting with sfGFP only, as reported in **Supplementary Table S2** and **Supplementary Figure S2**. Two multiparameter flow cytometry panels for the detection of antigen-specific B cell subsets and SARS-CoV-2–reactive T cells were used. Specifically, to determine the level of SARS-CoV-2–interacting B cells, the receptor binding domain (RBD) of SARS-CoV-2 S glycoprotein, fused to the superfolder green fluorescent protein (sfGFP) (18) was expressed and employed to identify the S/RBD-binding total and plasma B cells within the peripheral blood mononuclear cells (PBMCs) of all participants as previously reported (17).

Marker	Fluorochrome	Clone
CD3	APC	REA613
CD4	Vio® Bright B515	REA623
CD8	VioGreenTM	REA734
IFN-γ	PE	REA600
TNF-α	PE-Vio 770	REA656
CD14	VioBlue®	REA599
CD20	VioBlue	REA780
CD154 (CD40L)	APC-Vio 770	REA238
Live/dead	405/452	ViobilityTM Fixable Dye

Table S1. Panel of cell surface markers and fluorophore-conjugated antibodies used in the flow cytometry assay for the analysis of SARS-CoV-2–reactive T cells. APC, allophycocyanine; PE, phycoerythrin.

CD3	SB436	SK7
CD45	eFluor 506	HI30
CD19	PE	SJ25C1
CD38	PE-Cy5	HIT2
GFP	SARS-COV2-S-RBD	
Live/Dead	DRAQ7	

Table S2. Panel of cell surface markers and fluorophore-conjugated antibodies used in the flow cytometry assay for the analysis of SARS-CoV-2–reactive B cells. APC, allophycocyanine; PE, phycoerythrin.

	**Unexperienced No 151 (84.3%)**	**Covid experienced No 28 (15.6%)**	**p value**
Age, mean (SD), years<br>*Median (IQR)*	63.5 (10.8)<br>66 (57-71.7)	68.3 (9.6)<br>68 (65-75)	0.23
Sex: Male<br>*Female*	91 (60.3)<br>60 (39.7)	19 (76.0)<br>6 (24.0)	0.11

	Unexperienced<br>No 151 (84.3%)	Covid experienced<br>No 28 (15.6%)	p value
BMI, mean (SD)	25.9 (4.2)	27.7 (4.5)	0.38
Etiology of liver disease<br>*AIH/PBC/PSC*<br>*HBV/HDV/HCV*<br>*NAFLD*<br>*Alcohol abuse*<br>*Genetic hemocromatosis*	15 (9.9)<br>106 (70.1)<br>22 (14.5)<br>4 (2.6)<br>4(2.6)	1 (3.6)<br>21 (75.0)<br>4 (14.2)<br>1 (3.6)<br>1 (3.6)	0.86
CTP class<br>*A*<br>*B*<br>*C*	141 (93.3)<br>10 (6.6)<br>0	23 (84.0)<br>5 (16.0)<br>0	0.10
MELD	8.6 (2.8)	9.8 (4.1)	0.12
PLT	153.2 (77.9)	151.6 (75.7)	0.93
HCC y/n	13 (8.6)<br>138 (91.4)	1 (4.0)<br>24 (96.0)	0.00001
Oesophageal varices y/n	40 (26.5)<br>111(73.5)	11(39.2)<br>17 (60.7)	0.16
Day 7<br>*SARS-CoV-2-IgG level, mean(SD)*<br>*Median (IQR)*	190.1 (246.1)<br>1.50 (0.99-31.9)	893.25 (1489.3)<br>319.49(108.90-993.9)	<0.0001
Day 21<br>*SARS-CoV2-IgG level*<br>*Mean, (SD) BAU/ml*<br>*Median (IQR)*	296.2 (515.3)<br>150.0 (71.0- 273.3)	429.9 (308.0)<br>140.0 (101.8-473.1)	0.7
Day 31<br>*SARS-CoV2-IgG level*<br>*Mean, (SD) BAU/ml*<br>*Median (IQR)*	838.9 (864.8)<br>573.0 (193.4-1118.7)	1436.1 (1592.4)<br>858 (263.8-1798.4)	0.09

	Unexperienced No 151 (84.3%)	Covid experienced No 28 (15.6%)	p value
Day 90 SARS-CoV2-IgG level Mean, (SD) BAU/ml Median (IQR)	395.0 (440.5)<br>245.5 (132.5-410.4)	920.1 (1058.14)<br>412.8 (146.24-1115.2)	0.001
Day 180 SARS-CoV2-IgG level Mean, (SD) BAU/ml Median (IQR)	267.2 (221.5)<br>215.0 (111.7-334.8)	350.3 (216.6)<br>378.6 (59.1-436.1)	0.23

Table S3. Characteristics of patients without prior SARS-CoV-2 infection versus SARS-CoV-2 experienced cirrhotic patients

	Day	Cirrhotics - Unexperienced (Mean)	Cirrhotics - Covid Experienced (Mean)	SEM	Unexp vs. COV (pValue)

**B cells**	0	0.02631	0.8217	± 0.3011	0.0459
	7	0.02060	1.083	± 0.1129	0.0111
	21	0.08074	1.417	± 0.2884	0.0435
	60	0.1468	1.500	± 0.1758	0.0139
	180	0.1076	1.325	± 0.1297	0.0493
	Post-b	0.5600	1.250	± 0.3753	0.1334
**Plasma B cells**	0	0.03083	1.150	± 0.4559	0.0576
	7	0.01783	1.143	± 0.1670	0.0213
	21	0.08320	0.7467	± 0.1579	0.0515
	60	0.1534	1.697	± 0.1906	0.0128
	180	0.1384	1.420	± 0.1287	0.0332
	Post-b	0.6567	1.478	± 0.4832	0.1699
**CD4+ T cells**	0	0.02224	0.3745	± 0.1547	0.0717
	7	0.01895	0.7667	± 0.03336	0.0020
	21	0.03471	0.6333	± 0.1768	0.0762
	60	0.08052	0.8500	± 0.08266	0.0094
	180	0.07640	0.8000	± 0.1050	0.0639

	Post-b	0.3127	0.6458	± 0.1836	0.1396
**CD8+ T cells**	0	0.02511	0.4650	± 0.1950	0.0736
	7	0.01472	0.7040	± 0.1488	0.0436
	21	0.1280	0.5067	± 0.2476	0.2609
	60	0.07490	0.7333	± 0.1866	0.0695
	180	0.1044	0.6300	± 0.1385	0.1194
	Post-b	0.4640	0.7150	± 0.2136	0.2863

Table S4. Comparison between SARS-CoV-2 experienced and unexperienced cirrhotic patients. In the table, we report the means of cell fraction for each indicated subsets as well as the P values, generated by the comparison between experienced and unexperienced cohorts at the indicated time point. SEM, Standard Error of the Mean.

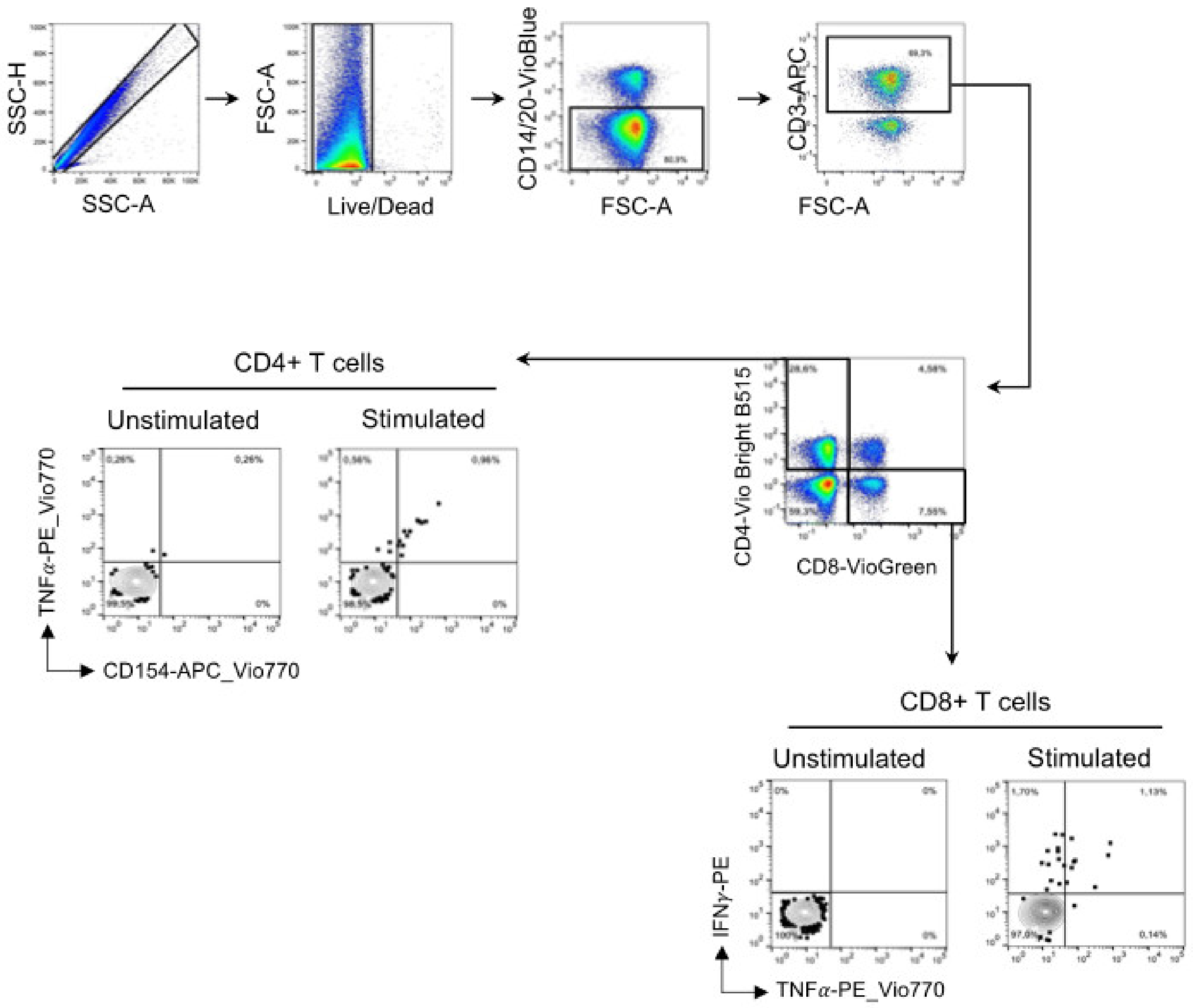

Figure S1. Overview of gating strategy for identifying the different T-cell subsets in peripheral blood mononuclear cells (PBMCs) after in vitro antigen stimulation. Fluorescence minus one (FMO) controls were used to set up all gates. Singlets were initially discriminated on SSC-H and SSC-A, followed by the exclusion of non-viable cells with Live/Dead violet fluorescent DNA dye and the identification of CD15-CD20- cell fraction. CD4+ and CD8+ T cells were identified after pre-gating on CD3+ cell fraction. Activated CD4+ T cells were defined as CD40 ligand (CD154)+ and TNFα+ cells, while activated CD8+ T cells were reported as TNF-α + and IFN-γ + cells.

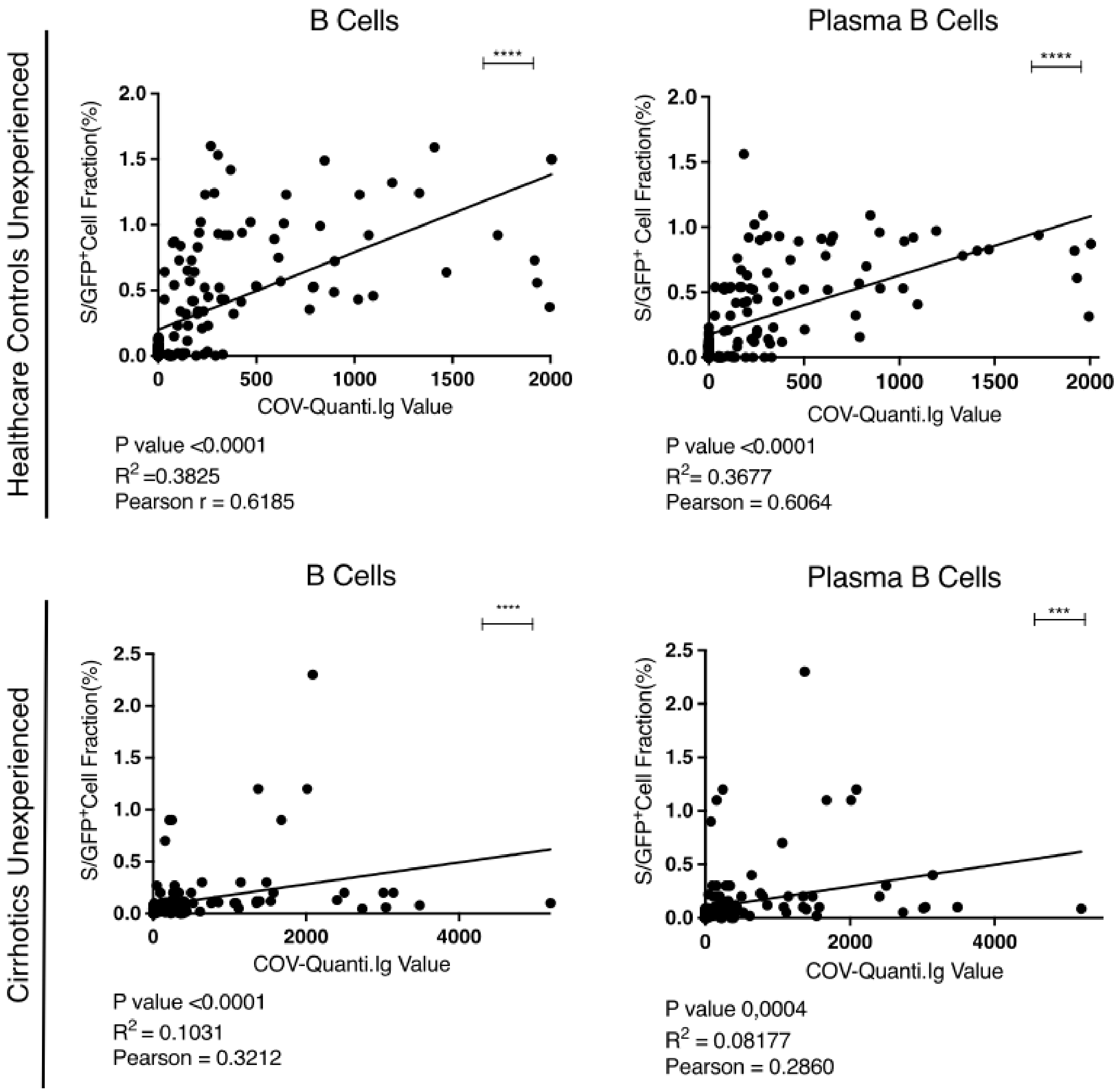

Figure S2. Correlation analysis between the abundance of indicated cell subsets, interacting with SARS-CoV-2 S RBD protein (S/GFP+ cells) and the COV-Quanti Ig value in all vaccinated participants. Pearson correlation coefficient (r), Spearman's rank correlation coefficient (ρ) and their statistical significance (P-value) are reported in the graphs. Linear correlation was evaluated trough the linear regression model. The linear regression line in black and R squared (R^2) are also shown in the graphs.
